# Structure and Function of Cyanobacterial DHDPS and DHDPR

**DOI:** 10.1038/srep37111

**Published:** 2016-11-15

**Authors:** Janni B. Christensen, T. P. Soares da Costa, Pierre Faou, F. Grant Pearce, Santosh Panjikar, Matthew A. Perugini

**Affiliations:** 1 Department of Biochemistry and Genetics, La Trobe Institute for Molecular Science, La Trobe University, Bundoora, Victoria 3086, Australia; 2 Biomolecular Interaction Centre and School of Biological Sciences, University of Canterbury, Christchurch 8140, New Zealand; 3 Australian Synchrotron, Clayton, Victoria 3168, Australia; 4 Department of Biochemistry and Molecular Biology, Monash University, Clayton, Victoria 3800, Australia

## Abstract

Lysine biosynthesis in bacteria and plants commences with a condensation reaction catalysed by dihydrodipicolinate synthase (DHDPS) followed by a reduction reaction catalysed by dihydrodipicolinate reductase (DHDPR). Interestingly, both DHDPS and DHDPR exist as different oligomeric forms in bacteria and plants. DHDPS is primarily a homotetramer in all species, but the architecture of the tetramer differs across kingdoms. DHDPR also exists as a tetramer in bacteria, but has recently been reported to be dimeric in plants. This study aimed to characterise for the first time the structure and function of DHDPS and DHDPR from cyanobacteria, which is an evolutionary important phylum that evolved at the divergence point between bacteria and plants. We cloned, expressed and purified DHDPS and DHDPR from the cyanobacterium *Anabaena variabilis*. The recombinant enzymes were shown to be folded by circular dichroism spectroscopy, enzymatically active employing the quantitative DHDPS-DHDPR coupled assay, and form tetramers in solution using analytical ultracentrifugation. Crystal structures of DHDPS and DHDPR from *A. variabilis* were determined at 1.92 Å and 2.83 Å, respectively, and show that both enzymes adopt the canonical bacterial tetrameric architecture. These studies indicate that the quaternary structure of bacterial and plant DHDPS and DHDPR diverged after cyanobacteria evolved.

Lysine is synthesised *de novo* in bacteria, plants and some fungi^1^,^2^,^3^. This occurs through either the α-aminoadipate pathway in fungi^4^ or the diaminopimelate pathway in bacteria and plants^1^,^2^,^3^. The diaminopimelate pathway commences with the condensation of pyruvate and (*S*)-aspartate semialdehylde (ASA) to form (4*S*)-4-hydroxy-2,3,4,5-tetrahydro-(2*S*)-dipicolinic acid (HTPA) (Fig. 1A)^1^,^2^,^3^,^5^,^6^. This reaction is catalysed by dihydrodipicolinate synthase (DHDPS) (EC4.3.3.7), which is the product of an essential gene in bacteria^1^,^2^,^7^,^8^,^9^. HTPA is subsequently non-enzymatically dehydrated to dihydrodipicolinate (DHDP), which is then reduced by dihydrodipicolinate reductase (DHDPR) (EC1.17.1.8) to form tetrahydrodipicolinate (THDP) (Fig. 1A)^1^,^10^,^11^. The pathway then diverges into four sub-pathways, namely the acetylase, aminotransferase, dehydrogenase and succinylase pathways, which operate across different genera and kingdoms^2^,^12^,^13^. For example, the aminotransferase pathway is canonical to plants, but is also innate to cyanobacteria, including *Anabaena variabilis (Kyoto Encyclopedia of Genes and Genomes* www.genome.jp/kegg-bin/show_module?ava_M00527+Ava_3607, 2016). The structure of DHDPS has been determined from a number of bacterial species, including *Agrobacterium* tumefaciens^14^, *Bacillus anthracis*^15^,^16^, *Escherichia coli*^17^,^18^, *Legionella pneumophila*^19^, *Mycobacterium tuberculosis*^20^, *Pseudomonas aeruginosa*^21^, *Staphylococcus aureus*^22^,^23^, *Streptococcus pneumoniae*^9^,^19^ and *Thermotoga maritima*^24^. The canonical bacterial DHDPS structure is a homotetramer best described as a ‘head-to-head’ dimer-of-dimers with four identical (β/α)_8_–barrel subunits (Fig. 1B)^3^,^7^,^25^. The structures of DHDPS from several plant species have also been determined showing that the plant orthologues also form homotetramers but in a ‘back-to-back’ dimer-of-dimers arrangement (Fig. 1B)^26^,^27^,^28^,^29^. Additionally, there are also reports of dimeric DHDPS enzymes from *P. aeruginosa*^21^, *S. aureus*^22^,^23^, and *Shewanella benthica*^30^.

Similarly, the structure of DHDPR has been determined from several bacterial species, including *Corynebacterium glutamicum*^31^, *E. coli*^32^,^33^, *M. tuberculosis*^34^,^35^ and *S. aureus*^36^. These studies show that the enzyme exists as a homotetramer with a unique quaternary architecture. Each monomer is comprised of an amino (N)-terminal NAD(P)H-binding domain that adopts a Rossman fold^37^, and a carboxyl (C)-terminal tetramerisation domain with the substrate-binding pocket formed between the N- and C-terminal domains (Fig. 1C). However, the structure of a plant DHDPR has not yet been determined, but a recent study employing small angle X-ray scattering suggests that the enzyme from *Arabidopsis thaliana* adopts a novel dimeric structure (Fig. 1C)^28^. Accordingly, there appears to be structural diversity between bacterial and plant orthologues of both DHDPS and DHDPR.

In this study, we aimed to determine the structure and function of the first DHDPS and DHDPR enzymes from a cyanobacterial species. Given that endosymbiotic theory suggests that the chloroplasts of plants were derived from the symbiosis of separate single bacterial cells^38^, we were interested in characterising the structure and function of DHDPS and DHDPR from the model cyanobacterial species, *Anabaena variabilis* (Av)^39^. Here, we present an in-depth characterisation of the structure and function of Av-DHDPS and Av-DHDPR in both solution and crystal states. We show that Av-DHDPS and Av-DHDPR both adopt the canonical bacterial structures, suggesting that the point of quaternary structural divergence between the bacterial and plants enzymes occurred after cyanobacteria evolved.

## Results and Discussion

### Purified recombinant Av-DHDPS and Av-DHDPR are active and folded

Av-DHDPS and Av-DHDPR were expressed in *E. coli* as His-tagged constructs and purified to >98% homogeneity using immobilised metal affinity chromatography (IMAC) (Fig. 2). The specific activity of purified Av-DHDPS and Av-DHDPR were determined to be 8.81 and 66.7 U/mg, respectively (Table 1), which correlate well to previous studies of recombinant orthologs^10^,^40^. MS/MS sequencing following trypsin digestion demonstrates that both recombinant *A. variabilis* enzymes are comprised of the correct primary structure (Table 1). CD spectroscopy was subsequently employed to demonstrate that recombinant Av-DHDPS (Fig. 3, open symbols) and Av-DHDPR (Fig. 3, solid symbols) contain 45–51% α/β structure, which is consistent with previous studies of bacterial and plant orthologues^7^,^9^,^14^,^16^,^19^,^22^,^25^,^27^,^41^.

### Enzyme kinetic properties

**Figure 1:**
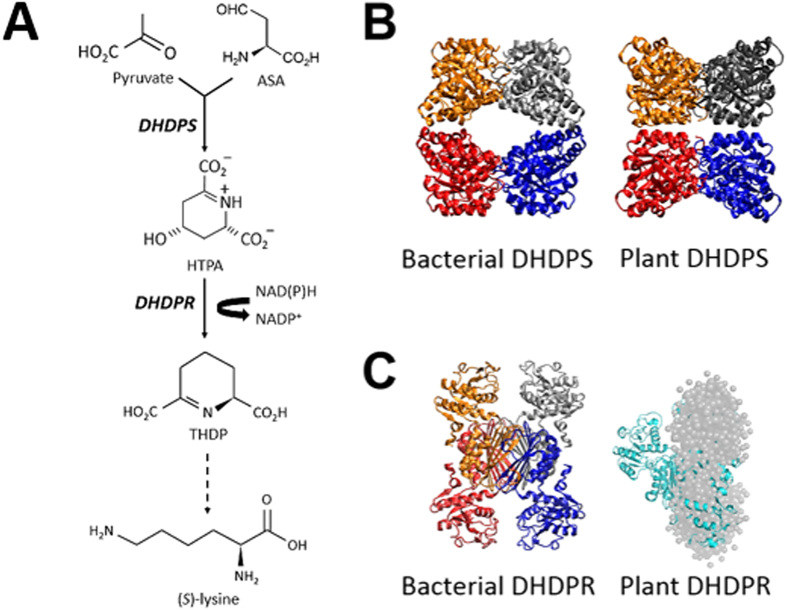
Diaminopimelate biosynthesis pathway. (**A**) The pathway commences with the condensation of pyruvate and ASA catalysed by DHDPS to produce HTPA. HTPA is then non-enzymatically dehydrated to yield DHDP, which is subsequently reduced by DHDPR to form THDP. THDP is converted into the final product (*S*)-lysine in a series of enzymatic steps utilising 4 different sub-pathways. (**B**) The three dimensional structures of bacterial DHDPS (PDB ID: 1YXC) and plant DHDPS (PDB ID: 3TUU). (**C**) Crystal structure of bacterial DHDPR (PDB ID: 1DIH) and the DAMMIN model of A. thaliana DHDPR^28^ (grey spheres) overlaying the structure of bacterial DHDPR (cyan) overlaid with the DAMMIN model of *A. thaliana* DHDPR^28^.

**Figure 2:**
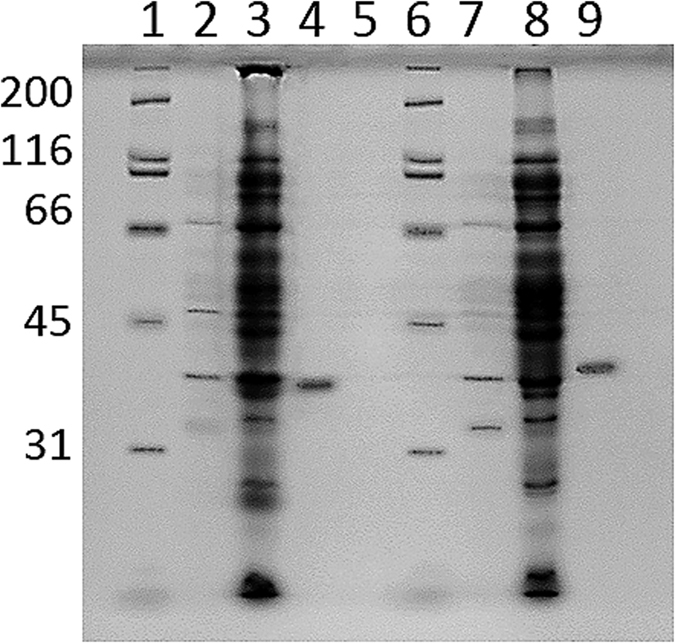
SDS-PAGE analyses of recombinant Av-DHDPS and Av-DHDPR. Lanes 1 & 6: molecular weight markers, kDa; lane 2: supernatant of non-ITPG treated cultures transformed with pRSETA-*dapA* (i.e. noninduced); lane 3: crude lysate post IPTG treatment of pRSETA-*dapA* transformed *E. coli* BL21-DE3 pLysS cells (i.e. induced); lane 4: post-IMAC purified recombinant Av-DHDPS; lane 7: supernatant of non-IPTG treated cultures transformed with pRSETA-*dapB*; lane 8: crude lysate post IPTG treatment of pRSETA-*dapB* transformed *E. coli* BL21-DE3 pLysS cells; lane 9: post-IMAC purified recombinant Av-DHDPR.

**Figure 3:**
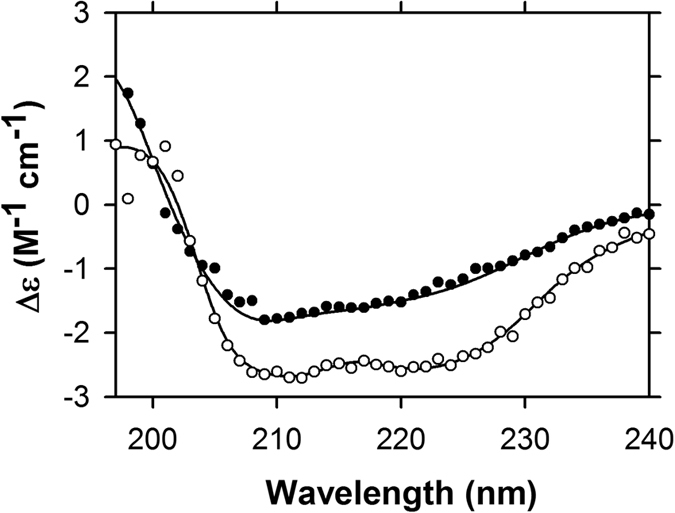
CD spectroscopy of recombinant Av-DHDPS and Av-DHDPR. Spectra were recorded using a protein concentration of 0.15 mg ml^−1^ between wavelengths of 195–240 nm employing a step size of 1.0 nm with 4 s averaging time. Raw data for Av-DHDPS (○) and Av-DHDPR (●) were fitted by nonlinear regression using the CDPro software and the CONTINLL algorithm (―), resulting in 33% α-helix, 18% β-structure, 14% β-turn and 35% unordered structure for Av-DHDPS with a RMSD of 0.070, and 18% α-helix, 27% β-structure, 13% β-turn and 42% unordered structure for Av-DHDPR with a RMSD of 0.037.

**Figure 4:**
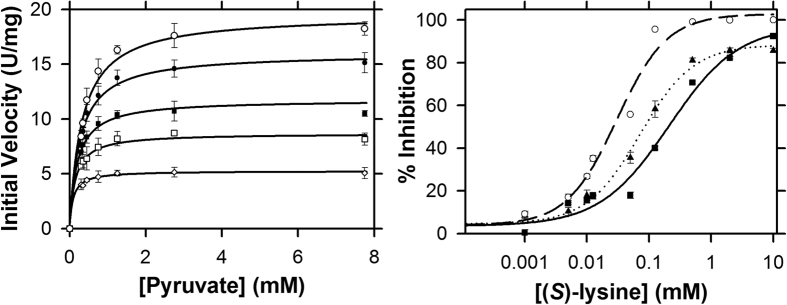
Enzyme kinetic profiles of recombinant Av-DHDPS. (A) Michaelis-Menten analyses of Av-DHDPS. The initial velocity is plotted as a function of pyruvate concentration at varying ASA concentrations of 0.0375 mM (◇), 0.075 mM (□), 0.125 mM (■), 0.25 mM (●), 0.5 mM (○). The global nonlinear best-fit using the ENZFITTER software (Biosoft) was obtained to a bi-bi ping pong model without substrate inhibition and resulted in *R*^2^ = 0.991. Data are presented as mean and error bars as standard deviation (*n* = 3). (B) Dose response curve showing initial rate plotted as a function of (*S*)-lysine concentration, for Av-DHDPS (▲), Ec-DHDPS (■), and Vv-DHDPS (○). The data were fitted to a ligand binding, four-parameter logistic function using ENZFITTER yielding *R*^2^ = 0.994 for Av-DHDPS (∙∙∙∙), *R*^2^ = 0.982 for Ec-DHDPS (──) and *R*^2^ = 0.982 for Vv-DHDPS (—). Data are presented as mean and error bars as standard deviation (*n* = 3).

Having determined that the recombinant cyanobacterial enzymes were homogenous, folded and enzymatically-active, we next set out to quantify their enzyme kinetic properties. Firstly, we characterised Av-DHDPS. Plots of initial rate as a function of varying pyruvate and ASA concentrations reveal typical Michaelis-Menten hyperbolic relationships (Fig. 4A). These data were globally fitted to yield a best fit to a bi-bi ping-pong mechanism without substrate inhibition (*R*^2^ = 0.99), providing the kinetic parameters summarised in Table 2. The resulting kinetic parameters agree well with previous studies of bacterial orthologues^9^,^14^,^16^,^20^,^22^,^24^. To establish whether recombinant Av-DHDPS is sensitive to allosteric feedback inhibition by (*S*)-lysine, which is the end product of the diaminopimelate pathway (Fig. 1A), enzyme assays were also performed with increasing (*S*)-lysine concentrations^1^,^2^,^3^,^18^,^19^,^29^. DHDPS from *E. coli* (Ec-DHDPS) and *V. vinifera* (Vv-DHDPS) were used as controls, given that previous studies show these orthologues are allosterically inhibited by (*S*)*-*lysine^18^,^29^. The dose-response curves for Av-DHDPS, Ec-DHDPS and Vv-DHDPS are shown in Fig. 4B with the nonlinear best fits to a four-parameter logistic function yielding an *IC*_50_^LYS^ = 0.068 mM (*R*^2^ = 0.99) for Av-DHDPS (Table 2), which is closer to the value obtained for Vv-DHDPS [*IC*_50_^LYS^ = 0.030 mM (*R*^2^ = 0.98)] than for Ec-DHDPS [*IC*_50_^LYS^ = 0.210 mM (*R*^2^ = 0.98)]. Interestingly, a recent study revealed that the amino acid at position 56 (*E. coli* numbering) determines whether DHDPS enzymes will be inhibited by (*S*)-lysine^19^. Moreover, a His or Glu at this position is a marker of allosteric inhibition, whereas DHDPS sequences that contain Lys or Arg at position 56 are insensitive to lysine-mediated allosteric inhibition. For Av-DHDPS, there is a Glu at position 58 (equivalent to position 56 in Ec-DHDPS), which is consistent with the recently established determinants of allostery for DHDPS enzymes^19^.

**Figure 5:**
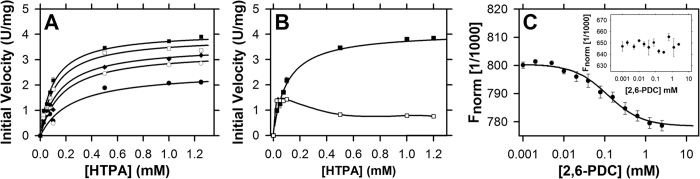
Kinetic parameters and cofactor preference of Av-DHDPR. (**A**) The initial rate of Av-DHDPR is plotted against increasing NADH concentrations of 0.0375 mM (●), 0.075 mM (○), 0.125 mM (◆), 0.25 mM (○) and 0.5 mM (■). The (─) represent the nonlinear best fit to a ternary complex mechanism without substrate inhibition using the ENZFITTER software (Biosoft), resulting in a *R*^2^ of 0.986. Data is presented as mean ± SD (*n* = 3). (**B**) The initial rate is plotted against either 0.2 mM [NADH] (■) or [NADPH] (○) with trend lines shown as solid lines (─). Data is presented as mean ± SD (*n* = 3). (**C**) Microscale thermophoresis binding curve of 2,6-PDC to Av-DHDPR in the presence of 150 μM NADP^+^ using the signal from Thermophoresis + T-Jump (●). The solid line (─) represents the nonlinear best fit to the single binding site model to yield a K_D_ of 100 ± 8.20 μM. *Inset:* 2,6-PDC titrations in the presence of 150 μM NAD^+^. Data are presented as mean and error bars as standard error (*N* = 3).

For Av-DHDPR, the enzyme kinetic parameters were determined employing *E. coli* DHDPS as the coupling enzyme using increasing DHDP and NADH concentrations (Fig. 5A). The nonlinear least squares global fit was obtained to a ternary complex model (*R*^2^ = 0.98), yielding the kinetic values reported in Table 2. A comparison of NADH and NADPH showed that Av-DHDPR is inhibited by its substrate, DHDP, when NADPH is employed as the cofactor (Fig. 5B). Subsequent thermodynamic measurements using microscale thermophoresis^19^,^42^ revealed that the cyanobacterial enzyme binds the substrate analogue 2,6-pyridinedicarboxylate (2,6-PDC)^34^ only when NADP^+^, and not NAD^+^, is present in the titration (Fig. 5C). This suggests that Av-DHDPR is inhibited by DHDP in the presence of the oxidised phosphorylated cofactor, which is consistent with *S. aureus* DHDPR^11^.

### Av-DHDPS and Av-DHDPR are tetramers in solution

**Figure 6:**
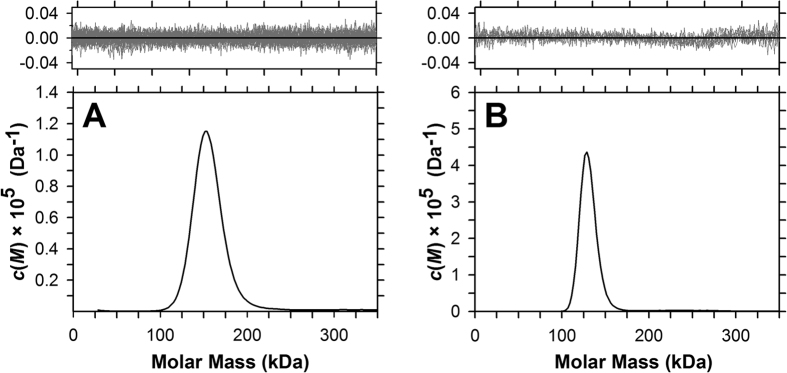
Analytical ultracentrifugation sedimentation velocity analyses. The *c(M*) distribution is plotted as a function of molar mass (kDa) for (**A**) recombinant Av-DHDPS and (**B**) recombinant Av-DHDPR. Top: Residuals plotted as a function of radial position resulting from the *c(M*) distribution best fit using N = 200 and P = 0.95, which yielded a rmsd = 0.00951 and runs test Z = 1.94 for Av-DHDPS and a rmsd = 0.00870 and runs test Z = 2.12 for Av-DHDPR.

To characterise the quaternary structure of Av-DHDPS and Av-DHDPR in solution, sedimentation velocity experiments were conducted in the analytical ultracentrifuge. The absorbance versus radial position profile for Av-DHDPS and Av-DHDPR at initial protein concentrations in the range of 0.1–7.0 μM show a distinct sedimentation boundary profile consistent with the presence of a single species (data not shown). Continuous size-distribution analyses of the data at 4.0 μM reveal that Av-DHDPS (Fig. 6A) and Av-DHDPR (Fig. 6B) have molar masses of 147 and 139 kDa with standardised sedimentation coefficients (*s*_20,w_) of 7.1S and 6.9S, respectively (Table 3). This indicates that both enzymes exist as stable tetramers in solution^16^,^24^,^41^,^43^. Additionally, the calculated *f*/*f*_0_ and axial ratio values (Table 3) are consistent with the asymmetric structures previously reported for DHDPS and DHDPR enzymes^16^,^24^,^41^,^43^.

### Crystal structures of Av-DHDPS and Av-DHDPR

**Figure 7:**
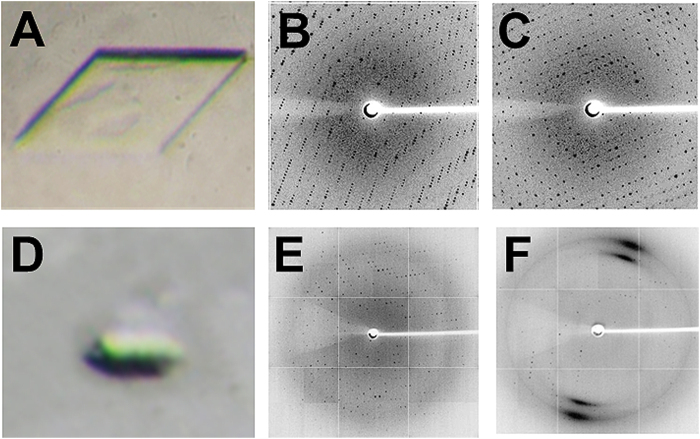
Crystallisation and X-ray diffraction data of recombinant Av-DHDPS and Av-DHDPR. (**A**) Photograph of Av-DHDPS crystal that was used to generate the diffraction patterns shown in (**B**) 90° rotation and (**C**) 180° rotation. (**D**) Photograph of Av-DHDPR crystal used to generate the diffraction patterns shown in (**E**) 90° rotation and (**F**) 180° rotation. Diffraction patterns were obtained using the MX2 beamline at the Australian Synchrotron as reported in the Methods section.

Av-DHDPS (7 mg/ml) was crystallised at 281 K in 16% (w/v) PEG3350, 0.4 M trisodium citrate, 0.1 M bis-Tris propane chloride, 10 mM pyruvate, pH 6.5, yielding crystals with dimensions of 0.1 mm × 0.05 mm (Fig. 7A). These crystals diffracted to a highest resolution of 1.92 Å (Fig. 7B,C). Similarly, Av-DHDPR (6 mg/ml) was crystallised at 281 K in 21% (w/v) PEG3350, 0.2 M lithium sulphate, 0.1 M bis-Tris chloride, pH 5.5, yielding crystals with dimensions of 0.04 mm × 0.015 mm (Fig. 7D) that diffracted to 2.83 Å (Fig. 7E,F). The diffraction data were subsequently used to determine the three-dimensional structure of Av-DHDPS and Av-DHDPR by molecular replacement.

**Figure 8:**
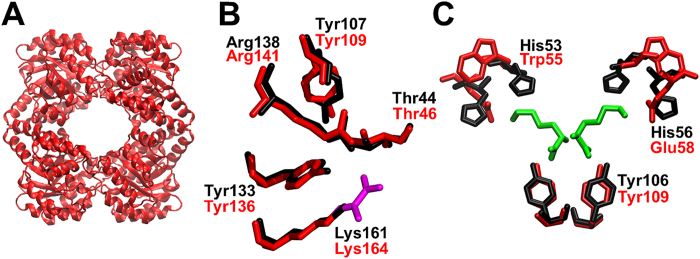
Av-DHDPS structure determined by X-ray crystallography. (**A**) Structure of Av-DHDPS (PDB ID: 5KTL) was solved to a resolution of 1.92 Å. (**B**) The active site of Av-DHDPS (red, PDB ID: 5KTL) overlayed with Ec-DHDPS (black, PDB ID: 1YXC). Shown in purple is the substrate pyruvate bonded to Lys164 in Av-DHDPS. (**C**) The allosteric site of Av-DHDPS (PDB ID: 5KTL) overlayed with Ec-DHDPS (black, PDB ID: 1YXD), with the allosteric inhibitor (*S*)-lysine shown in green (PDB: 1YXD).

Consistent with the analytical ultracentrifugation (AUC) studies in solution, the crystal structure of Av-DHDPS reveals a homotetramer that resembles the ‘head-to-head’ dimer-of-dimers canonical to bacterial orthologues (Fig. 8A). Each monomer is comprised of 297 residues that folds to form a N-terminal (β/α)_8_–barrel domain and a C-terminal domain consisting of 3 α-helices. Close inspection of the active site shows that it is comprised of the key conserved residues known to be important for catalysis, namely Lys164, Thr46, Tyr109, Tyr136 and Arg141, which are equivalent to Lys161, Thr44, Tyr107, Tyr133 and Arg138 in *E. coli* DHDPS (Fig. 8B)^17^,^18^. Likewise, inspection of the allosteric site confirms the presence of Glu at position 58 (His56 in Ec-DHDPS), but also reveals that Trp occupies position 55 (His53 in Ec-DHDPS), which is common in plants but not bacteria (Fig. 8C)^26^,^27^,^28^,^29^. The presence of a Trp at this positon is likely to explain the plant-like lysine *IC*_50_ for Av-DHDPS (Fig. 4B, Table 2).

**Figure 9:**
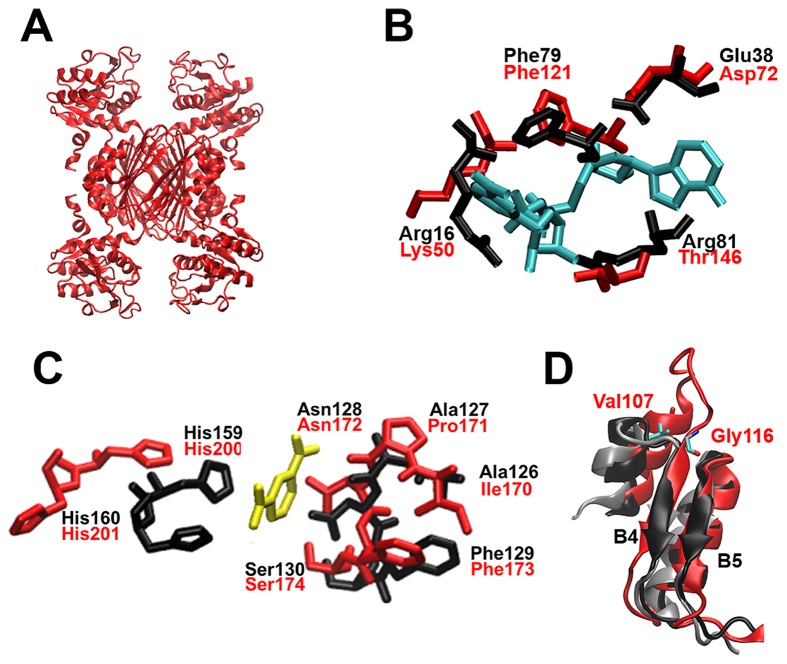
Av-DHDPR structure determined by X-ray crystallography. (**A**) Structure of Av-DHDPR (PDB ID: 5KT0) was determined to a resolution of 2.83 Å. (**B**) Nucleotide binding site of Av-DHDPR (red, PDB ID: 5KT0) overlayed with Ec-DHDPR (black, PDB ID: 1ARZ). Shown in cyan is the substrate NADH (PDB ID: 1ARZ). (**C**) Substrate binding site of Av-DHDPR (red, PDB ID: 5KT0) overlayed with Ec-DHDPR (black, PDB ID: 1ARZ). Shown in yellow is the substrate analogue 2,6-PDC (PDB ID: 1ARZ). (**D**) Extended loop in Av-DHDPR (red) between residues Val107 and Gly116 located between β-sheets B4 and B5 overlayed with Ec-DHDPR (grey, PDB ID: 1ARZ) and *M. tuberculosis* DHDPR (black, PDB ID: 1YL5).

The Av-DHDPR crystal structure also reveals a homotetramer (Fig. 9A), which is consistent with the in-solution AUC studies, and agrees well with previously characterised bacterial DHDPR structures^31^,^32^,^33^,^34^,^35^,^36^. Each monomeric unit consists of 287 residues with an N-terminal nucleotide and a C-terminal tetramerisation domain connected via a hinge region. Although residue variation is observed at the nucleotide binding site, the physicochemical properties of the residues are still conserved (Fig. 9B) as observed for other bacterial DHDPR species^31^,^32^,^33^,^34^,^35^,^36^. By contrast, the substrate binding cleft of Av-DHDPR is predominantly conserved (Fig. 9C). However, Av-DHDPR has a unique prolonged solvent-exposed loop located between β-sheets B4 and B5, consisting of residues Val107 to Gly116 (Fig. 9D). Overall, the loop residues have a neutral, slightly hydrophobic nature, and the function of the loop is unclear. This prolonged loop is absent in all other published DHDPR structures suggesting this is a unique feature of cyanobacterial DHDPR^31^,^32^,^33^,^34^,^35^,^36^.

### Bioinformatics analysis

**Figure 10:**
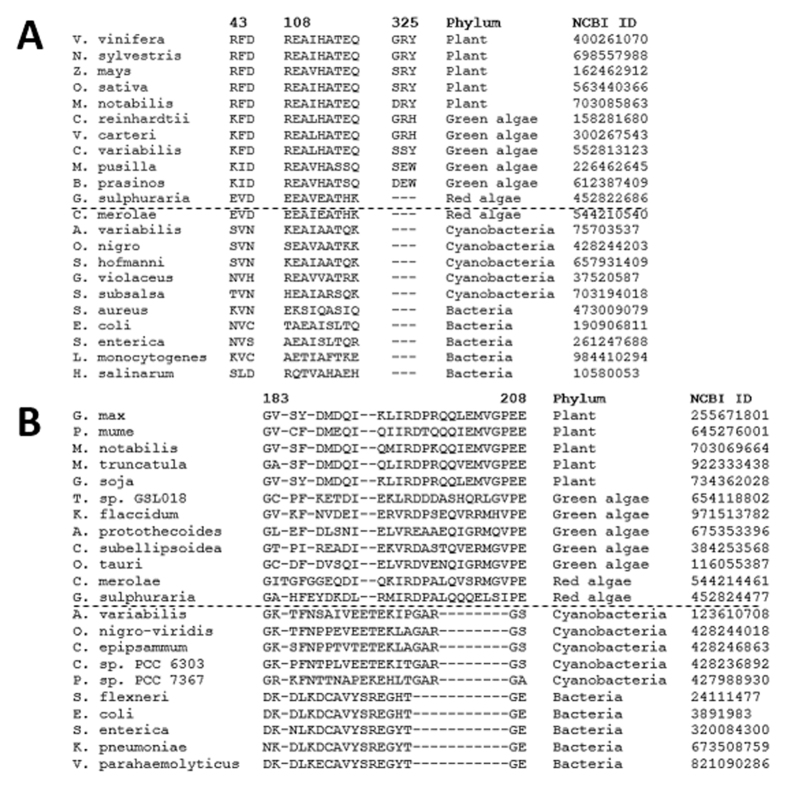
Bioinformatics sequence analyses of DHDPS and DHDPR in multiple phyla. (**A**) Multiple sequence alignment of the DHDPS tetramerisation interface residues (*V. vinifera* DHDPS numbering). The sequence motifs 43–45, 108–116 and 325–327 are conserved in plants and green algae but absent in bacteria and cyanobacteria. (**B**) Multiple sequence alignment of the flexible loop motif (183–204, *E. coli* numbering) in DHDPR. The motif length is significantly shorter in bacteria and cyanobacteria compared to red algae, green algae and plants. The long dashed line in panels A and B indicates the point of structural divergence based on sequence similarity, whereas individual dashes (−) represent a gap in the sequence. For simplicity, this alignment only shows a fraction of the sequences analysed.

This study reveals that both Av-DHDPS and Av-DHDPR adopt the canonical bacterial tetrameric architecture. This was unexpected given the endosymbiosis theory^38^ and the shared aminotransferase pathway found in both cyanobacteria and plants. Consequently, bioinformatics sequence analyses of DHDPS and DHDPR from several bacterial and plant species were performed to predict when the plant structures first evolved. For DHDPS, the dataset employed consisted of sequences from 150 bacteria, 85 cyanobacteria, 84 plants, 12 green algae and 2 red algae from the NCBI protein database (www.ncbi.nlm.nih.gov/protein, 2016). A representative subset of these sequences are aligned in Fig. 10A. It was noted that the motifs Arg43 to Asp45, Arg108 to Gln116 and Gly325 to Tyr/His327 (*V. vinifera* numbering) are conserved in plants and form an interacting network at the tetramerisation interface in plant structures^26^,^27^,^28^,^29^. These motifs were also found in green algae but not in bacteria, cyanobacteria or red algae species (Fig. 10A). This finding suggests that the point of divergence of the DHDPS quaternary structures occurred between red and green algae. This remains to be verified experimentally.

To examine whether the same pattern is observed for DHDPR, sequences from 25 bacteria, 57 cyanobacteria, 38 plants, 12 green algae and 2 red algae from the NCBI protein database were obtained (July 2016). Figure 10B shows a representative multiple sequence alignment of a subset of these species. The length of the loop motif in cyanobacteria (Gly183-Ser203, *E. coli* DHDPR numbering) is similar in length to other bacterial species (Fig. 10B). However, this loop is significantly longer in plant, red algae and green algae sequences (Fig. 10B). This suggests that DHDPR from red and green algae may adopt a similar dimeric quaternary architecture to plant orthologues. This also remains to be verified experimentally.

## Conclusions

In this study, we determined for the first time the enzyme kinetic parameters, solution properties and three-dimensional structures of DHDPS and DHDPR from a cyanobacterial species. We show both enzymes exist as homotetramers in solution and in the crystal state, and that they adopt the canonical bacterial quaternary architecture. Our results suggest that the point of structural divergence differentiating bacterial and plant DHDPS and DHDPR enzymes occurred between cyanobacteria and lower plants.

## Methods

### Cloning, expression and purification of Av-DHDPS and Av-DHDPR

Synthetic codon-optimised Av-DHDPS (i.e. *dapA*) and Av-DHDPR (i.e. *dapB*) genes cloned into pRSET-A expression vectors were purchased from GeneArt. The plasmids were subsequently transformed into *E. coli* BL21-DE3 pLysS cells for the overexpression of the recombinant enzymes. Recombinant protein was produced by treating *E. coli* BL21-DE3 pLys cells with 1.0 mM IPTG at 25 °C for 8 h. Cells were harvested by centrifugation (5000 × g) and resuspended in 20 mM Tris-HCl, pH 8.0, 500 mM NaCl, 20 mM imidazole, 5% (*v*/*v*) glycerol, which included 10 mM pyruvate for Av-DHDPS, given that pyruvate is known to stabilise DHDPS enzymes^16^,^22^. The cell suspension was lysed on ice by sonication using a Vibra Cell VC40 (Sonics & Materials) at 40 micron using 6 cycles of 10 sec on followed by 2 min off. Recombinant His-tagged enzymes were isolated from the cell lysate using IMAC employing a 5 ml His-Trap column (GE Healthcare) and a 0–500 mM imidazole linear gradient over 17 column volumes. Av-DHDPS and Av-DHDPR eluted at 195 mM and 140 mM imidazole, respectively. The purified protein was dialysed overnight against 20 mM Tris-HCl, pH 8.0, 100 mM NaCl, 5% (*v*/*v*) glycerol, which included 10 mM pyruvate for Av-DHDPS.

### Tandem mass spectrometry

Purified recombinant Av-DHDPS and Av-DHDPR were subjected to trypsin digestion and tandem mass spectrometry (MS/MS) sequencing using a Thermo Scientific LTQ Orbitrap Elite ETD Mass Spectrometer as previously reported^44^,^45^.

### Circular dichroism spectroscopy

Circular dichroism (CD) spectra of Av-DHDPS and Av-DHDPR were obtained using an Aviv Model 420 CD spectrometer using similar methods reported previously^14^,^16^,^22^,^27^,^41^,^46^. Briefly, wavelength scans were performed between 195 and 240 nm with a 4.0 sec averaging time in 20 mM Tris, pH 8.0, 150 mM NaCl (also containing 1 mM pyruvate for Av-DHDPS) in a 1.0 mm quartz cuvette. Data were analysed using the CDPro software package incorporating the SP22X database^47^,^48^.

### DHDPS-DHDPR coupled enzyme kinetic assay

Kinetic analyses of Av-DHDPS and Av-DHDPR were performed employing the DHDPS-DHDPR coupled assay as previously described^7^,^9^,^11^,^14^,^16^,^19^,^22^,^24^,^25^,^27^,^28^,^29^. Briefly, assays were performed in triplicate at 30 °C in a 1 cm acrylic cuvette using *E. coli* DHDPR and *E. coli* DHDPS as the coupling enzymes for Av-DHDPS and Av-DHDPR, respectively. Mixtures were allowed to equilibrate in a temperate-controlled Cary 4000 UV-Vis spectrophotometer for 12 min before initiating the reaction with ASA. The initial reaction rate data were analysed using the ENZFITTER software (Biosoft). Data were fitted to various models, including the bi-bi ping-pong and ternary complex models with and without substrate inhibition, with best fits determined from the highest *R*^2^ value.

### Microscale thermophoresis

Affinity measurements using microscale thermophoresis (MST) were carried out with a Monolith NT. LabelFree instrument (NanoTemper Technologies)^19^,^42^. 2,6-PDC diluted in water (5.0 mM to 2.4 μM) was mixed 1:1 with the enzyme pre-incubated with 150 μM NAD^+^ or NADP^+^, yielding a final DHDPR concentration of 10 μM and a dilution series of 2.5 mM to 1.2 μM of 2,6-PDC. Controls were performed in the absence of NAD^+^/NADP^+^, with water added instead, but with the same dilution series for 2,6-PDC. All experiments were incubated for 30 min at 30 °C, before applying samples to Monolith NT Standard Treated Capillaries (NanoTemper Technologies). Thermophoresis was measured at 30 °C with 5 s/30 s/5 s laser off/on/off times. Experiments were conducted at 30% LED power and 40% MST IR-laser power. Data from three independently performed experiments were fitted to the single binding site model (NT. Analysis software version 1.5.41, NanoTemper Technologies) using the signal from Thermophoresis + T-Jump.

### Analytical ultracentrifugation

Sedimentation velocity experiments were performed at 20 °C in a Beckman Coulter Model XL-A analytical ultracentrifuge using double sector quartz cells and An50-Ti rotor^49^,^50^. 400 μl of buffer and 380 μl of sample at an initial concentration ranging from 0.1 μM to 7.0 μM were loaded into the reference and sample sectors of the cells, respectively. The rotor was accelerated to 40,000 rpm and data were collected continuously at 230 nm using a step size of 0.003 cm without averaging. Initial scans were carried out at 3,000 rpm to determine the optimal wavelength and radial positions for the high speed experiment. Solvent density, solvent viscosity, and estimates of the partial specific volume of Av-DHDPS (0.736 ml/g) and Av-DHDPR (0.736 ml/g) at 20 °C were calculated using SEDNTERP^51^. Data were fitted using the SEDFIT software (www.analyticalultracentrifugation.com) to a continuous size-distribution model^52^,^53^,^54^,^55^.

### Crystallisation and X-ray diffraction

Av-DHDPS and Av-DHDPR were crystallised using the hanging-drop vapour diffusion method as described previously^10^,^30^,^40^,^56^. For X-ray data collection, crystals were transferred to reservoir solution containing 20% (*v*/*v*) glycerol and 12.5 mM MnCl_2_, and directly flash frozen in liquid nitrogen. Intensity data were collected at the Australian Synchrotron using the MX2 beamline. Diffraction data were processed using MOSFLM^57^ and scaled using SCALA^58^. Molecular replacement was performed using the MR protocol of Auto-Rickshaw^59^ with *B. anthracis* DHDPS (PDB ID: 1XKY) as the search model for Av-DHDPS and *M. tuberculosis* DHDPR (PDB ID: 1YL5) as the search model for Av-DHDPR. Structural refinement was performed using REFMAC5^60^ with iterative model building using COOT^61^. The refinement statistics are provided in Table 4. For Av-DHDPS, Ramachandran statistics showed 91.3% in the preferred region, 8.3% in the additionally allowed region and 0.4% in the disallowed region consistent with previous structural reports^15^,^21^,^62^. For Av-DHDPR, Ramachandran statistics showed 89.5% in the preferred region, 8.8% in the additionally allowed region and 1.3% in the generously allowed region consistent with previous studies^31^,^32^,^33^,^34^,^35^,^36^. However, 0.4% of residues (i.e. Ser89 and Gln112) were in the disallowed region due to poor electron density.

## Additional Information

**How to cite this article**: Christensen, J. B. *et al.* Structure and Function of Cyanobacterial DHDPS and DHDPR. *Sci. Rep.* **6**, 37111; doi: 10.1038/srep37111 (2016).

**Publisher’s note:** Springer Nature remains neutral with regard to jurisdictional claims in published maps and institutional affiliations.

## Figures and Tables

**Table 1:** Purification of recombinant Av-DHDPS and Av-DHDPR.

Enzyme	Purification step	Total activity (U)	Total protein (mg)	Specific activity (U mg^−1^)	Fold^1^	MS/MS coverage^2^
Av-DHDPS	Crude	179	144	1.24	—	45%
	IMAC	170	19.3	8.81	7.10	
Av-DHDPR	Crude	265	186	24.8	—	100%
	IMAC	93.2	69.2	66.7	2.68	

^1^Fold of purified enzyme post-IMAC relative to crude.

^2^Sequence coverage from MS/MS analyses post in-gel tryptic digestion.

**Table 2:** Enzyme kinetic parameters for recombinant Av-DHDPS and Av-DHDPR.

Enzyme	*K*_M_^PYR^	*K*_M_^ASA^	*k*_cat_	*IC*_50_^LYS^(mM)
Av-DHDPS	0.41	0.14	15	0.068
	***K***_**M**_^**DHDP**^	***K***_**M**_^**NADH**^	***k***_**cat**_	
Av-DHDPR	3.6	0.31	3.0	N/A

**Table 3:** Hydrodynamic properties of recombinant Av-DHDPS and Av-DHDPR.

Enzyme	*s*_20,*w*_ (S)^1^	Molar Mass (kDa)^2^	*f*/*f*_0_^3^	Axial ratio (a/b)^4^
Av-DHDPS	7.1	147	1.3	2.5
Av-DHDPR	6.9	139	1.3	2.6

^1^Standardised sedimentation coefficient calculated from SEDNTERP software.

^2^Determined from the ordinate maximum of the *c(M*) distribution best fits (Fig. 6).

^3^Frictional coefficient calculated from *s*_20,w_ using the ⊽ method employing SEDNTERP software.

^4^Calculated from SEDNTERP using a prolate model (⊽ method).

**Table 4:** X-ray data collection and refinement statistics for recombinant Av-DHDPS (PDB ID: 5KTL) and Av-DHDPR (PDB ID: 5KT0).

Parameter	Av-DHDPS	Av-DHDPR
Wavelength (Å)	0.9537	0.9537
No. of images	360	360
Oscillation angle per frame	0.5	1.0
Space group	P2_1_2_1_2	I222
Unit-cell parameters		
a, b, c (Å)	75.73, 154.35, 55.79	72.73, 89.36, 95.92
α, β, γ (°)	90, 90, 90	90, 90, 90
Resolution (Å)	1.92	2.83
Observed reflections	335888 (47075)	110053 (17182)
Unique reflections	49053 (7452)	7762 (1225)
Completeness (%)	99.1 (94.5)	99.7 (98.7)
*R*_merge_	10.3 (78.0)	10.0 (36.6)
Mean *I*/*σ(I*)	14.50 (2.68)	25.08 (8.36)
Redundancy	6.85 (6.32)	14.18 (14.03)
Molecules per asymmetric unit	2	1
Wilson-*B*	31.86	40.86
*R*_*work*_/*R*_*free*_ [%]	18.0/22.4	18.8/25.3
*R*_*free*_ test set count	1016	452
Protein/solvent/metal atoms	4413/228/5	2037/1/0
Average *B* factor [Å^2^]	50.6	43.8
Favored/additionally allowed/generously allowed/disallowed residues in Ramachandran plot [%]	91.3/8.3/0.0/0.4	89.5/8.8/1.3/0.4

Values in parentheses are for the highest resolution bin.
